# Glycyrrhizic Acid Improves Cognitive Levels of Aging Mice by Regulating T/B Cell Proliferation

**DOI:** 10.3389/fnagi.2020.570116

**Published:** 2020-10-07

**Authors:** Ruichan Jiang, Jiaming Gao, Junyan Shen, Xiaoqi Zhu, Hao Wang, Shengyu Feng, Ce Huang, Haitao Shen, Hailiang Liu

**Affiliations:** ^1^Key Laboratory of Xinjiang Phytomedicine Resource and Utilization of Ministry of Education, College of Life Sciences, Shihezi University, Shihezi, China; ^2^Shanghai East Hospital, Institute for Regenerative Medicine, Tongji University School of Medicine, Shanghai, China

**Keywords:** glycyrrhizic acid, learning and memory, cognition, T cells, B cells

## Abstract

Glycyrrhizic acid (GA) is the substance with the highest content of triterpenoid saponins that can be extracted from licorice, and has anti-inflammatory, neuroprotective, and anticancer functions, among others. The aim of this study was to investigate the protective effect of GA on cognitive decline in middle-aged mice and explore its mechanisms. We injected GA by the tail vein of C57BL/6 mice and measured their cognitive levels using the Morris water maze. The Morris water maze results demonstrated that GA improved learning and memory abilities in middle-aged mice. Furthermore, the RNA-sequencing and flow cytometric analyses revealed that GA could increase T and B cells. We then confirmed the relationship between cognition and the immune system in the immune-deficient B-NDG mouse model. Our results suggest that GA improves cognition in aging mice by regulating T/B cell proliferation.

## Introduction

Cognitive decline is a characteristic of human aging, and age-related deterioration of learning and memory also occurs in rats (Frick et al., 2000). Therefore, age-related decline in spatial memory has been extensively studied in rats. These studies have shown that deteriorations in spatial learning and memory functions can be observed after 4–5 months of age (Shoji et al., 2016), and these functions decline from 12 months of age in C57BL/6J mice (Bach et al., 1999). Therefore, preventing age-related decline in middle-aged mice has important applications for humans.

**FIGURE 1:**
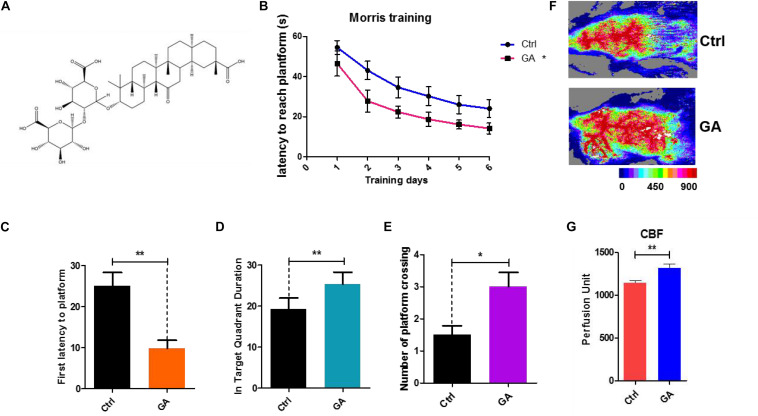
Glycyrrhizic acid ameliorated cognitive declines in middle-aged mice, improved cerebral blood flow. **(A)** The chemical structure of Glycyrrhizic acid (GA). **(B)** Escape latency of six consecutive daily tests. Statistical comparisons used the repeated measures ANOVA. **(C)** First latency to the platform in the probe trial. **(D)** Time spent in the target quadrant in the probe trial. **(E)** Crossing times of the target platform in the probe trial. **(F)** Images of CBF in C57BL/6. **(G)** Quantification of blood perfusion in C57BL/6. GA (5 mg/kg, once every other day); Data represent mean ± SEM (*n* = 8 per group). *P < 0.05, **P < 0.01, vs. Ctrl.

Licorice is the root of *Glycyrrhiza glabra* L. (Leguminosae), which grows in various warm climates such as the Middle East, Asia, and Southern Europe. It is one of the oldest known medicinal herbs and is referred to as “the father of herbal medicine.” Glycyrrhizic acid (GA) (Figure 1A), a triterpenoid saponin, is a major component of licorice. It has a variety of pharmacological activities such as anti-inflammatory, antioxidant, anticancer, neuroprotective, and immune-regulatory effects, among others (Wang et al., 2020). Previous studies indicated that GA produces robust neuroprotection via the modulation of anti-apoptotic and pro-apoptotic factors, primarily through the ERK signaling pathway and its anti-inflammatory properties against high-mobility group box 1 phosphorylation and the suppression of inflammatory cytokine induction (Gong et al., 2012; Kim et al., 2012; Teng et al., 2014). These results were based on pathological models. Although numerous pathways have been implicated in the neuroprotective effects of GA, the molecular mechanisms are not yet completely understood. In this study, we aimed to investigate the effects of GA in preventing age-related cognitive disorders, and the underlying molecular mechanisms.

## Materials and Methods

### Reagents

Glycyrrhizic acid (GA) was obtained from TAUTO (Sichuan, China). 4′,6-diamidino-2-phenylindole dihydrochloride (DAPI), lysis buffer, and all antibodies used for flow cytometry were purchased from BD Pharmingen (San Diego, CA). Information regarding the antibodies used in this study is listed in the Table 1.

**TABLE 1:** Resources information used in this study.

**Reagent or resource**	**Source**	**Identifier**
**Antibodies**		
CD3e	BD Biosciences	Cat# 553063
CD45R/B220	BD Biosciences	Cat# 553087
CD49b	BD Biosciences	Cat# 558295
CD4	BD Biosciences	Cat# 550954
CD8a	BD Biosciences	Cat# 563152
CD45	BD Biosciences	Cat# 566439
CD3	BD Biosciences	Cat# 560590
CD44	BD Biosciences	Cat# 553134
CD62l	BD Biosciences	Cat# 560516
CD45R/B220	BD Biosciences	Cat# 553087
CD138	BD Biosciences	Cat# 558626
CD27	BD Biosciences	Cat# 558754
**Chemicals**		
Glycyrrhizic acid	TAUTO	Cat# 1405-86-3
Lysing Buffer	BD Biosciences	Cat# 555899
DAPI	BD Biosciences	Cat# 564907
TRIzol	Gibco	Cat# 15596018
the Prime Script^TM^ RT Master Mix (Perfect Real Time)	Takara	Cat# RR036A
SYBR Green fluorescent dye	BioRad	Cat# 172-5120
PBS	Gibco	Cat# 10010049
PFA	Sigma	Cat# 158127
Nestin	Millipore	Cat# MAB5326
SOX2	Abcam	Cat# ab93689
**Experimental model**		
B-NDG	Biocytogen	B-CM-002
C57BL/6	SLACCAS	
**Oligonucleotides**		

**Gene**	**Forward primer**	**Reverse primer**

CD3e	CTGCTACACACCAG CCTCAA	GTAATAAATGACCATCA GCAAGC
CD45R/B220	CCAGTGATGCTACCA CAACG	CAATCCTCATTTCCACAC TTAGC
β-actin	TATTGGCAACGAGC GGTTC	ATGCCACAGGATTCCA TACCC

### Animals

Both 8-week-old and 8-month-old female SPF C57BL/6 mice were obtained from Shanghai SLACCAS Co., Ltd. (Shanghai, China). 8-week-old female SPF B-NDG mice were obtained from Jiangsu Biocytogen Co., Ltd. (Nantong, China). All mice were housed five per cage and maintained on a 12 h light/dark schedule and allowed free access to food and water following a protocol approved by the Animal Research Committee of Tongji University School of Medicine, China. GA was administrated by tail vein every other day for 42 days. Control mice were tail vein administrated the same volume of PBS.

### qRT-PCR Analysis

Total RNA was isolated using TRIzol reagent (Thermo Fisher Scientific, Waltham, MA, United States), and cDNA was prepared using the Prime Script^TM^ RT Master Mix (Perfect Real Time) (Takara, Dalian, China) according to the manufacturer’s protocols. The qRT-PCR reactions were performed using SYBR green fluorescent dye (BioRad). Primer sequences are listed in the Table 1.

### The Morris Water Maze Task

The Morris water maze consists of a round, black pool of 120 cm in diameter and 31 cm deep containing water at 23 ± 1°C. The escape platform (11 cm in diameter, adjustable height) was placed in the center of one quadrant of the pool and hidden below the water surface at 1.5 cm deep. Various prominent visual cues were placed around the pool and remained in the same position during training and testing periods. Each group was trained for 6 consecutive days from four locations and then tested on day 6 with a new direction, removing the hidden platform and allowing free swimming for 60 s. The escape latencies from the water, and the distance traveled to find the platform was recorded using video-animal tracking software.

### Novel Object Recognition

Each mouse was habituated to an empty novel object recognition (NOR) open-field box for two 10 min test sessions 24 h apart. Twenty-four hours after the last habituation session, mice were subjected to training during a 10 min exposure session of two identical, non-toxic objects (metal or hard plastic items) in the open-field box. The time spent exploring each object was recorded using ObjectScan software (Clever Sys. Inc., Reston, VA); an area 2 cm^2^ surrounding the object was defined, so that nose entries within 2 cm of the object was recorded as time exploring the object. After the training session, the animal was returned to its home cage. After a retention interval of 1 h (Zhang et al., 2016), the animal was returned to the arena in which two objects, one identical to the familiar object but previously unused (to prevent olfactory cues and prevent the necessity of washing objects during experimentation) and one novel object. The animal was allowed to explore for 10 min, during which the amount of time exploring each object was recorded. Objects were randomized and counterbalanced across animals. Animals that spent <7 s exploring the objects during the 10 min test session were omitted from the analysis. Objects and arenas were thoroughly cleaned with 70% isopropanol between trials. For the novel object recognition tests, the time spent exploring the novel object (familiar vs. novel) was reported as the recognition index, and was calculated using the following formula: [(time exploring specified object)/(time exploring novel object + time exploring familiar object)] × 100 (Taglialatela et al., 2009). Statistical analysis of NOR data is done by first performing an one-sample *t-*test to determine if the mean percentage exploration time is significantly different from a theoretical mean of 0.5000. This is followed by one-way ANOVA to determine group differences (Hernandez et al., 2010).

### Open Field Test

The device is based on a square field (50 × 50 × 30 cm). A lamp was located 150 cm above the field, and the illumination of the central area was approximately 100 lux. At the beginning of each experiment, a mouse was placed in a 15 × 15 cm central area. At the beginning of the experiment, each mouse was placed in the center of the field, and the time spent in the central area within 10 min was recorded.

### Immune Reconstitution (IR)

Harvested mouse spleens were digested and washed with phosphate buffered saline (Thermo Fisher Scientific, Waltham, MA, United States). Then, splenocytes were obtained by removing the red blood cells with lysis buffer (BD Biosciences, San Jose, CA, United States) and filtering through a 70 μm cell strainer (Jet Biofil, Guangzhou, China). Then, 3 × 10^5^ splenocytes were administered via the tail vein (McDermott et al., 2010).

### Cortical Cerebral Blood Flow Measurements

Images were acquired with a laser speckle contrast imager (PeriCam PSI System, Perimed, Stockholm, Sweden). We used the PeriCam PSI HD system to calculate an arbitrary index of cerebral blood flow (perfusion units) in the ipsilateral hemisphere.

### Cell Preparation and Flow Cytometric Analysis

Harvested mouse spleens were macerated and washed with phosphate buffer saline (Thermo Fisher Scientific, Waltham, MA, United States). Then, splenocytes were obtained by removing the red blood cells with lysis buffer (BD Biosciences, San Jose, CA, United States) and filtering through a 70 μm cell strainer (Jet Biofil, Guangzhou, China). Additionally, fresh blood samples were collected in heparinized tubes. T (CD3^+^) cells, B (CD3^–^CD45R/B220^+^) cells, and NK (CD3^–^CD49b^+^) cells in the spleen and blood were directly quantified using flow cytometry (Beckman FC-500, Miami, FL, United States).

Some splenocytes were cultured with added GA in 6-well plates for 36 h. Then effector T (CD45^+^CD3^+^CD44^+^CD62l^–^) cells, and effector B (CD45^+^CD3^–^CD45R/B220^+^CD138^+^CD27^+^) cells were directly quantified using flow cytometry. All antibodies were used at an optimized working concentration of 1 μg/ml.

### Immunofluorescence Images

Brain tissue was collected from mice following treatment with GA. Brain tissue was fixed with 4% paraformaldehyde (PFA) in 0.1 M phosphate-buffered saline (PBS) overnight, followed by a 15–30% sucrose gradient dehydration for 1 day until the brain had completely sunk to the bottom of the sucrose solution. An optimal cutting temperature compound was used to embed tissue samples. Successive coronal sections of 10 μm were cut using a freezing microtome. Tissue slices were washed with PBS and blocked for 1 h (in 10% bovine serum albumin, 3% normal donkey serum, and 1% Triton X-100 in PBS) and mounted on tissue slides. Antibodies against Nestin and SOX2 (Abcam, Cambridge, MA, United States), which can be used to label quiescent radial-glia-like type I neural progenitor cells (Encinas et al., 2011), were used. The next day, the slides were washed three times and incubated with the appropriate Alexa 488- and Alexa 568- secondary antibodies (Thermo Fisher Scientific) for 1 h at room temperature (1:1,000 dilution). DAPI staining was used to label nuclei. Slides were examined using an OLYMPUS BX53 microscope (Olympus, Madison, WI).

### RNA Sequencing Analysis

RNA sequencing was performed independently and uniformly for each sample. GA- and control-treated mice were anesthetized and euthanized, and two blood samples were removed for RNA-seq following extraction of total RNA. Clean reads were aligned to the reference gene sequence using bowtie-2, and the gene expression levels of each sample were calculated. DEG detection was conducted using the DEGseq method. The statistical results were based on the ma-plot method. The number of reads of specific genes obtained from the sample was sampled randomly, and then *P*-values were calculated according to the normal distribution and corrected to *q*-values. To improve the accuracy of DEG detection, genes with a difference multiple of more than twice, and a *q* ≤ 0.001 were screened and defined as significantly differentially expressed genes.

### Statistical Analysis

Statistical analysis of data was conducted using Graphpad Prism 5.0 and expressed as the mean ± standard error of the mean (SEM). Statistical comparisons of two groups were made using the unpaired *t*-test. Escape latency of six consecutive daily tests used the repeated measures ANOVA. Probability values less than 0.05 were considered statistically significant.

## Results

### GA Prevented Impairments of Learning and Memory Displayed and Improved CBF in Middle-Aged C57BL/6 Mice

Twelve-month-old female C57BL/6 mice were injected with GA (5 mg/kg, once every other day, tail intravenous injection, i.v.) for 6 weeks. Then, we tested the behavioral effects of GA using the Morris water maze (MWM) task to evaluate spatial learning and memory. The MWM showed that the GA-treated mouse latencies to the platform gradually decreased compared with the control after 6 days of training (Figure 1B). In the training test, the test results for the main effect of time, age are respectively, *F* = 45.34, *P* < 0.001 and *F* = 4.569, *P* = 0.0451. The interaction effects were not statistically significant (*P* = 0.6212). The impairment in learning and memory displayed in middle-aged mice can be prevented by GA treatment as indicated by decreased escape latencies (Figure 1C), more time in the target quadrant (Figure 1D), and increased platform crossings (Figure 1E). This effect was accompanied by improved cerebral blood flow (CBF) (Figures 1F,G). Both the control and GA groups exhibited similar swim speeds to the virtual platform (Supplementary Figure S1). These data suggested that GA improved CBF and prevented impairments of learning and memory displayed in middle-aged C57BL/6 mice.

### GA-Treatment Actived T/B Genes Expression in the Blood RNA-Seq

**FIGURE 2:**
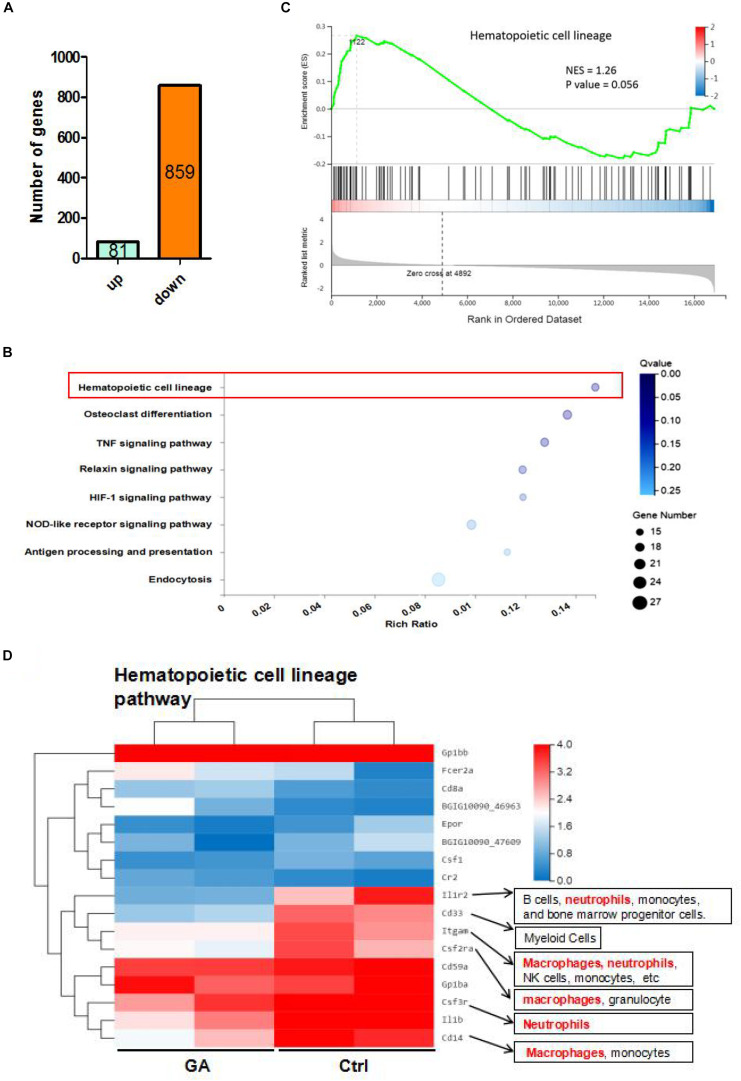
The RNA-seq results of GA and control mice. **(A)** The differential expressed genes between GA and control group. **(B)** Enrichment plots of gene expression signatures for hematopoietic cell lineage. Barcode plot indicates positions of genes in each gene-set. NES, normalized enrichment score. **(C)** Bubble diagram showing GA treatment influence the hematopoietic cell lineage. **(D)** Heatmap showing differentially expressed genes (DEGs) in hematopoietic cell lineage of control and GA-treated C57BL/6 mice. There were two different representative mice from each treatment group.

We analyzed the transcriptome of C57BL/6 mice blood using RNA-seq. There were 940 differentially expressed genes between the GA and control groups, of which 81 were upregulated and 859 were downregulated (Figure 2A). KEGG pathway analysis also demonstrated that GA significantly influenced the hematopoietic cell lineage (Figure 2B). Furthermore, gene set enrichment analysis of these differentially expressed genes revealed that GA treatment activated the hematopoietic cell lineage (Figure 2C). The differential genes in the pathway were clustered using heat mapping (Figure 2D). The RNA-seq results showed that GA activated CD8a, Fcer2a (CD23), and Cr2 (CD21) expression and that it inhibits the expressions of macrophage and neutrophil-related genes.

### GA-Treatment Increased T/B Cell Numbers in the Blood and Spleen

**FIGURE 3:**
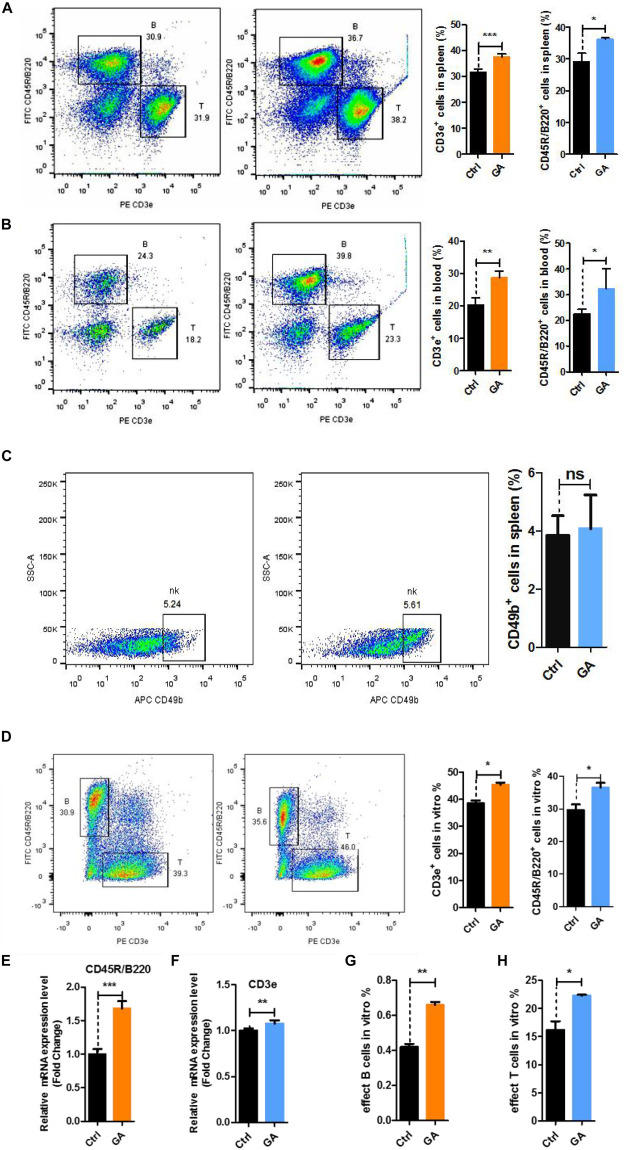
GA increased the proliferation of T and B cell subsets *in vitro* and *vivo*. **(A)** Representative FACS plots showing CD3e^+^ T and CD45R/B220^+^ B cells in the spleen of C57BL/6 mice; Bar graphs for statistical results of T and B cells in spleen of C57BL/6 mice. **(B)** Representative FACS plots showing CD3e^+^ T and CD45R/B220^+^ B cells in the blood of C57BL/6 mice. **(C)** Representative FACS plots showing CD49b^+^ NK cells in the spleen of C57BL/6 mice. **(D)** Representative FACS plots showing CD3e^+^ T and CD45R/B220^+^ B cells *in vitro*. **(E)** CD3e mRNA expression in spleen of C57BL/6 mice. **(F)** CD45R/B220 mRNA expression in spleen of C57BL/6 mice. **(G)** Bar graphs for statistical results of effect B cells after flow cytometry sorting. **(H)** Bar graphs for statistical results of effect T cells after flow cytometry sorting. Data represent mean ± SEM (*n* = 8 per group). *P < 0.05, **P < 0.01, ***P < 0.001. ns, not significant, vs. Ctrl.

To further verify the relationships of the active genes to cognitive ability, we analyzed the levels of CD3e^+^ T cells, CD45R/B220^+^ B cells, and CD49b^+^ NK cells in the blood and spleen after 42 days of GA treatment. Flow cytometry results showed that GA could increase T and B cell numbers in the blood and spleen (Figures 3A,B), whereas NK cell numbers were unchanged in the spleen (Figure 3C).

To demonstrate that GA produces the similar effects *in vitro*, we used GA-treated single cells obtained from 8-week-old C57BL/6 mouse spleens for 36 h and measured the changes of T and B cells. Flow cytometry showed that GA could significantly increase the numbers of T and B cells *in vitro* (Figure 3D). Quantitative real-time PCR showed that *CD3e* and *CD45R/B220* genes are overexpressed (Figures 3E,F). We treated T cells and B cells obtained from 8-week-old C57BL/6 mouse spleens using flow cytometry sorting with 50 μM GA and respectively measured effector T cells (CD45^+^CD3^–^CD45R/B220^+^CD138^+^CD27^+^) and effector B cells (CD45^+^CD3^+^CD44^+^CD62l^–^). The results showed that GA could increase effector cells *in vitro* (Figures 3G,H). These results showed that GA increased T and B cells *in vivo* and *in vitro*.

### GA Improved Cognitive Levels by Regulating T/B Cell Proliferation

**FIGURE 4:**
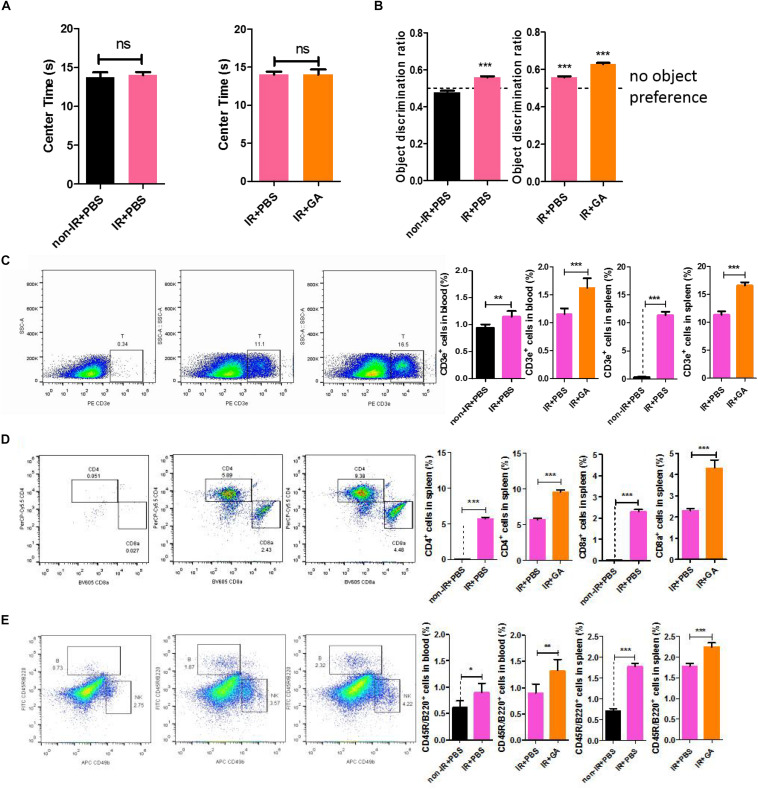
GA increased the speed of immunological reconstitution in B-NDG mice and enhanced the cognitive ability. **(A)** Detection of anxiety using open-field test. Results are expressed the time spent in the center area. **(B)** IR + PBS and IR + GA mice showed a preference for the novel object after a 1 h delay between identical object exploration during training and the introduction of a novel object during the test phase. *T*-test against a theoretical mean of 0.5000 yielded *p*-values of 0.0005 and <0.0001 for IR + PBS and IR + GA, respectively. Non-IR + PBS not exhibits any preference for object exploration (*p* = 0.051). *** indicates significant difference at *p* < 0.0001 from theoretical mean of 0.50. **(C)** Representative FACS plots showing CD3e^+^ T cells in the spleen of B-NDG mice; Bar graphs for statistical results of T cells in blood and spleen of B-NDG mice. **(D)** Representative FACS plots showing CD4 and CD8 T cells in the spleen of B-NDG mice; Bar graphs for statistical results of CD4 and CD8 T cells in spleen of B-NDG mice. **(E)** Representative FACS plots showing CD45R/B220^+^ B and CD49b^+^ NK cells in the spleen of B-NDG mice; Bar graphs for statistical results of B cells in blood and spleen of B-NDG mice. GA (5 mg/kg/2 days); Data represent mean ± SEM (*n* = 8 per group). *P < 0.05, **P < 0.01, ***P < 0.001, ns, not significant, vs. Ctrl.

To further investigate whether the neuroprotective effects of GA were related to the T and B cells, we used B-NDG (NOD-*Prkd^*scid*^ IL2rg^*tm1*^*/Bcgen) mice (Supplementary Figure S2), which lack T, B, and NK cells. We tested behavioral effects after immune reconstitution (IR) and IR + GA treatment by performing a novel object recognition task to measure the attention and non-spatial declarative memory in B-NDG mice. Before the novel object recognition task, we performed the open field test to evaluate the anxiety behavior of these mice. Anxiety levels can be determined based upon the time the mice remain in the corner of the enclosure. We found that the ratio of the time the IR + PBS and IR + GA mice spent in the center of the enclosure to the time spent in a corner over a 10 min test session was not significantly different compared with the control group (Figure 4A). The novel object recognition task showed that IR + PBS mice exhibited a significant preference for object exploration (Figure 4B). After IR, the GA treatment showed a more significant preference when contrasted with PBS treatment (Figure 4B).

We analyzed the levels of CD3e^+^ positive T cells, CD45R/B220^+^ positive B cells, and CD49b^+^ positive NK cells in the blood and spleen (Supplementary Figure S3). Flow cytometry results showed that the IRs of B-NDG mice were successful given that T, B, and NK cells in IR + PBS mice were significantly increased compared with controls (Figures 4C,E and Supplementary Figure S4). Thus, GA treatment after IR can increase the number of T and B cells compared with PBS treatment after IR (Figures 4C,E), whereas NK cell numbers were unchanged (Supplementary Figure S4). This result was consistent with those above from C57BL/6 mice. Furthermore, we measured the numbers of CD4 and CD8 cells in the spleen and found they were increased (Figure 4D), which was consistent with our RNA-sequencing results.

### GA-Treatment Increased Neural Stem Cells in the Dentate Gyrus

**FIGURE 5:**
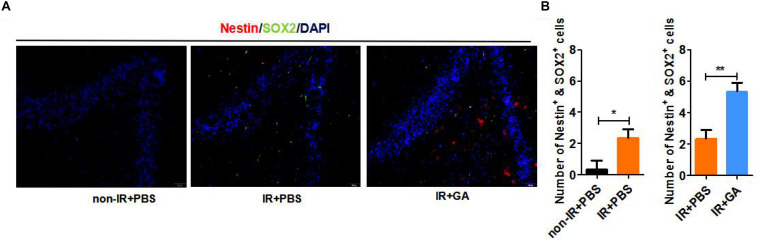
GA-treatment increased neural stem cells in the dentate gyrus. **(A)** Expression of Nestin and SOX2 neural stem cell marker in B-NDG mice dentate gyrus. **(B)** Quantitative analysis of the numbers of Nestin + SOX2 + double-positive cells in dentate gyrus. GA (5 mg/kg/2 days); Data represent mean ± SEM (*n* = 8 per group). *P < 0.05, **P < 0.01, vs. Ctrl.

To further investigate various mouse brain tissue characteristics, we examined the neural stem cell markers Nestin and SOX2 in the dentate gyrus (DG) of the hippocampus in IR + PBS, or IR + GA treated mice and control mice. The results showed that the numbers of Nestin + SOX2 + double-positive cells were significantly increased in the DG of immunologically reconstituted mice compared with the control and GA treatment, or compared with PBS injection after IR (Figures 5A,B).

## Discussion

Our work has thus revealed that GA treatment can revitalize the aging brain and alleviate aging-associated cognitive declines and that these effects may be related to the immune system. RNA-seq data demonstrated the effect of GA on hematopoietic cell lineage, and we posited that GA could enhance cognition through immune system modulation. We observed lymphocyte changes in C57BL/6 mice, then used B-NDG mice to verify further the importance of the immune system in cognitive ability. The B-NDG mice exhibit a severe immunodeficiency phenotype with no mature T cells, B cells, or functional NK cells and a lack of cytokine signaling ability. The results of the novel object recognition task confirmed our hypothesis; after IR, cognitive ability was improved (Figure 4B).

A marked increase in the number of cytotoxic CD4 T cells (CD4 cytotoxic T lymphocytes) is a signature characteristic of supercentenarians (Hashimoto et al., 2019). We observed that CD4^+^ T cells were significantly expanded in number (Figure 4D), but we cannot confirm whether these represent cytotoxic CD4 T cells. Although we did not investigate immune responses in the brain, many studies have demonstrated the role of CD4 T cells in neuroinflammatory and neurodegenerative processes (Brochard et al., 2009; Baruch et al., 2015; Dansokho et al., 2016). Pasciuto showed that absence of the CD4 T cell population resulted in microglia remaining suspended between a fetal and adult developmental state, resulting in defects in synaptic pruning function and normal mouse behavior (Pasciuto et al., 2020). Additionally, microglial regeneration was found to improve spatial learning ability and promote hippocampal nerve regeneration (Willis et al., 2020). We hypothesized that GA affects cognition by acting on immune cells in the blood and brain, further influencing microglia in the mouse brain, which requires further study.

The brain is particularly susceptible to the effects of aging, and aging-associated inflammation is a major risk factor for a variety of neurocognitive and neurodegenerative diseases (Fung et al., 2020). There are abnormal neural stem cells, neurons, and microbes in the brain, and their clearance by immune cells can preserve cognitive function (Bussian et al., 2018). Further efforts to understand the effector cells in this process and how these events occur would be worthwhile in understanding the roles of immune cells in brain aging.

During cytokine storms, cytokine levels are abnormally high, which can lead to fever, low blood pressure, and heart problems and, in some cases, organ failure and death (RIddell, 2018). Bacterial infection and viruses such as SARS (severe acute respiratory syndrome) and MERS (Middle East respiratory syndrome) can cause cytokine storms, which attack host cells, mainly the patient’s immune cells (Tisoncik et al., 2012). Macrophages and neutrophils are known to produce catecholamines in response to inflammatory stimuli such as lipopolysaccharide (LPS), which is a hallmark of many types of bacterial infection (Flierl et al., 2007). Our RNA-seq results showed that GA treatment could inhibit several genes related to macrophages, neutrophils, and IL1β (Supplementary Figure S1). This finding indicates that GA might inhibit the proliferation of macrophages and neutrophils from preventing cytokine storms. Treatment with GA can also alleviate β-amyloid (Ahn et al., 2006; Zhao et al., 2013) or systemic LPS-induced (Song et al., 2013) cognitive impairment via inhibition of neuroinflammation. Some COVID-19 patients have been alleviated symptoms using diammonium glycyrrhizinate, an ammonium salt preparation of 18-alpha-GA (Ding et al., 2020; Li et al., 2020). This phenomenon could be explained by the anti-inflammatory properties of GA. In conclusion, the potential effects of GA treatments on memory and behavior, longevity, and anti-viral activity may be worthy of future investigation.

## Data Availability Statement

The datasets presented in this study can be found in online repositories. The names of the repository/repositories and accession number(s) can be found below: https://www.ncbi.nlm.nih.gov/, GSE146239.

## Ethics Statement

The animal study was reviewed and approved by the Animal Research Committee of Tongji University School of Medicine, China.

## Author Contributions

RJ, JG, JS, XZ, HW, SF, and CH performed the experiments. RJ and HL analyzed the data and prepared the manuscript. HL and HS supervised the project. HL designed the project. All authors contributed to the article and approved the submitted version.

## Conflict of Interest

The authors declare that the research was conducted in the absence of any commercial or financial relationships that could be construed as a potential conflict of interest.

## Supplementary Material

Supplementary Figure 1 Mean speed was also not different between the two groups. Ctrl, Control; GA, Glycyrrhizic acid. The overall significance between two groups was determined by Student’s *t*-test. ns, not significant.

Supplementary Figure 2 The animal graphical abstract in B-NDG mice.

Supplementary Figure 3 Gating strategies for identifying T (including CD4 and CD8a cells), B and NK cells in B-NDG mice.

Supplementary Figure 4 Related to Figure 4E. Bar graphs for statistical results of NK cells in blood and spleen of B-NDG mice. ^∗∗∗^*P* < 0.001; ns, not significant.
