# Supplementary material for: Glycyrrhizic Acid Improves Cognitive Levels of Aging Mice by Regulating T/B Cell Proliferation
Source: Front Aging Neurosci. 2020 Oct 7;12:570116. doi: 10.3389/fnagi.2020.570116 (PMC7575738; doi:10.3389/fnagi.2020.570116)
Supplement: Supplementary Figure 1 — Mean speed was also not different between the two groups. Ctrl, Control; GA, Glycyrrhizic acid. The overall significance between two groups was determined by Student’s t-test. ns, not significant. [file Data_Sheet_1.doc]

**Figure S1. Mean speed was also not different between the two groups. Ctrl: Control; GA: Glycyrrhizic acid. The overall significance between two groups was determined by Student’s t-test. ns, not significant.**


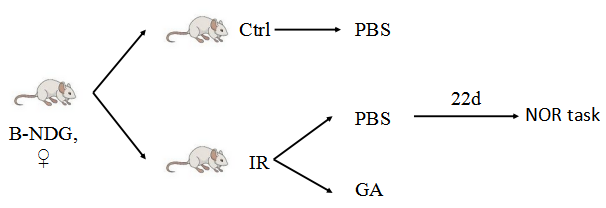


**Figure S2. The animal graphical abstract in B-NDG mice.**


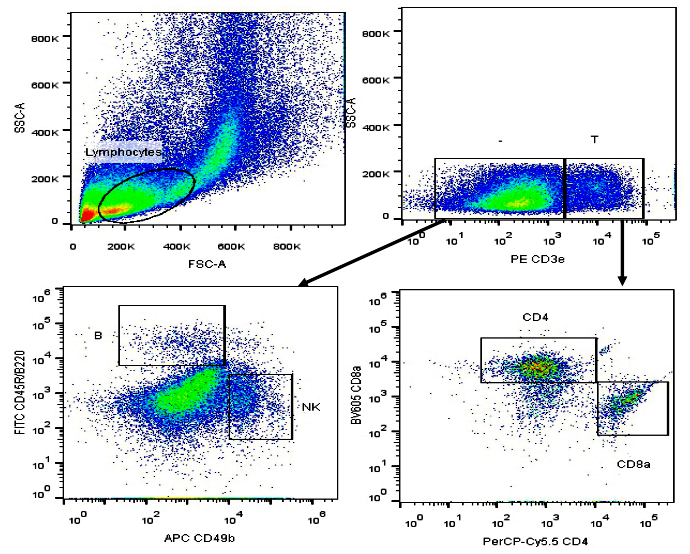


**Figure S3. Gating strategies for identifying T (including CD4 and CD8a cells), B and NK cells in B-NDG mice.**


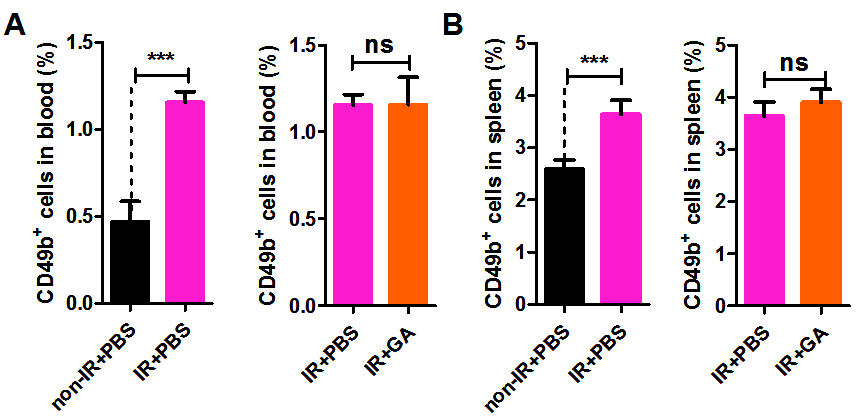


**Figure S4 (Related to Figure 4E). Bar graphs for statistical results of NK cells in blood and spleen of B-NDG mice. ***P < 0.001, ns, not significant.**
